# Die Messung von „Patient-reported Outcomes“ als Instrument patientenzentrierter Versorgung?

**DOI:** 10.1007/s00103-026-04187-w

**Published:** 2026-01-20

**Authors:** Matthias Rose

**Affiliations:** 1 Medizinische Klinik mit Schwerpunkt Psychosomatik, Centrum für Innere Medizin und Dermatologie, Charité – Universitätsmedizin Berlin, Charitéplatz 1, 10117 Berlin, Deutschland; 2 Center for Patient-Centered Outcomes Research CPCOR, Charité – Universitätsmedizin Berlin, Berlin, Deutschland; 3 Deutsche Gesellschaft für patientenberichtete Outcomes und Gesundheitsdaten e. V. DG-PRO, Berlin, Deutschland; 4 Health Outcomes Observatory H2O, Berlin, Deutschland; 5 Netzwerk Universitätsmedizin– Methoden- & Bioproben-Hub – NUM-MB – Standort Berlin, Würzburg, Deutschland; 6 Nationales Centrum für Tumorerkrankungen NCT – Standort Berlin, Berlin, Deutschland; 7 Deutsches Zentrum für Psychische Gesundheit DZPG – Standort Berlin, Berlin, Deutschland

**Keywords:** Symptom Monitoring, Real World Evidence (RWE), Klinische Praxis, Medizininformatik-Initiative (MII), Health Outcomes Observatory (H2O), Symptom monitoring, Real World Evidence (RWE), Clinical care, Medicine Informatics Initiative (MII), Health Outcomes Observatory (H2O)

## Abstract

Patient-reported Outcome Measures (PROMs) gewinnen zunehmend an Bedeutung für die patientenzentrierte Beurteilung des Behandlungserfolges im Gesundheitswesen. In den letzten Jahrzehnten wurde eine große Zahl von Instrumenten entwickelt, die eine differenzierte Erfassung von Symptomen, Alltagsfunktionen und verschiedenen Aspekten der Lebensqualität erlaubt. Bei annähernd 50 % aller klinischen Studien werden PROMs mittlerweile eingesetzt, um Behandlungen hinsichtlich ihres Nutzens für die Behandelten zu vergleichen. Zudem werden PROMs auch in der Qualitätssicherung zur Beurteilung des Behandlungserfolges verschiedener Gesundheitsanbieter herangezogen. Bei der individuellen Behandlung können PROMs dem Screening auf psychische Belastungen oder Erkrankungen dienen.

Relativ neu ist die Nutzung von PROMs als Intervention selber. Mit einer systematischen Symptomerfassung in der Onkologie lassen sich Nebenwirkungen besser steuern und regelhaft positive Effekte hinsichtlich der Lebensqualität der Patienten zeigen.

Dennoch werden PROMs in der klinischen Routine bislang wenig genutzt. Für die Anwender wiegt derzeit der Gewinn für deren klinische Arbeit den Mehraufwand bislang nur selten auf. Voraussetzungen für eine gelungene Implementierung in die klinischen Abläufe sind zum einen eine stärkere, transsektorale Harmonisierung der Messungen, zum anderen eine bessere Integration in die digitalen Informationssysteme. In Deutschland und Europa sind in den letzten Jahren verschiedene Initiativen entstanden, u. a. als Teil der Medizininformatik-Initiative, die sich mit beiden Themen beschäftigen. Es scheint kaum denkbar, dass eine empirisch orientierte Medizin langfristig auf die regelmäßige Erfassung eines der zentralen Therapieziele – wie es den Behandelten geht – verzichten wird.

## Abstract (en)

Patient-reported outcome measures (PROMs) are becoming increasingly important for patient-centered assessment of treatment success in healthcare. In recent decades, a large number of instruments have been developed that allow for differentiated recording of symptoms, everyday functions, and various aspects of quality of life. PROMs are now used in nearly 50% of all clinical studies to compare treatments in terms of their benefits for patients. In addition, PROMs are used in quality assurance to compare the treatment success of various healthcare providers. In individual treatment, PROMs can be used to screen for psychological burden or mental health condition.

The use of PROMs as an intervention in themselves is relatively new. Systematic symptom assessment in oncology allows side effects to be better controlled and regularly shows positive effects on patients’ quality of life.

Nevertheless, PROMs have been little used in routine clinical practice to date. For clinicians, the benefits for their daily work rarely outweigh the additional effort involved. The prerequisites for successful implementation in clinical processes currently appear to be, on the one hand, intersectoral harmonization of measurements and, on the other hand, better integration into electronic health records. In Germany and Europe, various initiatives have emerged in recent years, including as part of the Medical Informatics Initiative, which address both issues. It seems inconceivable that empirically oriented medicine will in the long term dispense recording one of the central therapeutic goals—how the patient is doing.

## Einleitung

Traditionell wird der Behandlungserfolg in der Medizin vor allem über Mortalitätsraten, Komplikationshäufigkeiten, Wiederaufnahmeraten oder ökonomische Kennzahlen bewertet. Diese Daten spiegeln jedoch kaum wider, wie Patienten die Versorgung bzw. die Ergebnisse ihrer Versorgung erleben. Mit dem demografischen Wandel und der Zunahme chronischer Erkrankungen wächst daher das Interesse an patientenzentrierten Messinstrumenten [1]. Zwei zentrale Ansätze hierfür sind die Erfassung von *Patient-reported Outcomes *(PROs) und *Patient-reported Experiences* (PREs). PROs und PREs werden häufig zusammen gemessen, um die Patientensicht aus beiden komplementären Perspektiven zu verstehen, wobei die Messung von PROs der Symptomerfassung und der Beurteilung der Ergebnisqualität dient, während die Erfassung der erlebten Versorgungsabläufe die Prozessverbesserung unterstützt.

Der Terminus PRO, der vor 2 Jahrzehnten von der Food and Drug Administration (FDA) mit geprägt wurde [2], ist in gewisser Hinsicht irreführend, weil *Patient-reported Outcome Measures* (PROMs) sowohl zur Erfassung psychosozialer Determinanten des Behandlungserfolges (*Screening*), zur Messung von Therapienebenwirkungen (*Monitoring*) als auch zur Beurteilung des Therapieerfolgs (*Outcome*) eingesetzt werden. PROMs können dabei krankheitsspezifisch (z. B. für Patienten mit Lungentumoren, Quality of Life Questionaire (QLQ) der European Organisation for Research and Treatment of Cancer (EORTC) Lung Cancer (LC) Module EORTC QLQ-LC13 [3]) bzw. therapiespezifisch sein (z. B. für Patienten unter Immune-Checkpoint-Inhibitor (ICI)-Therapie, EORTC QLQ-ICI) oder generische Aspekte der Gesundheit erfassen (z. B. Short Form-(SF-)36 [4]). In der Regel werden generische Instrumente mit erkrankungs- oder therapiespezifischen kombiniert.

Der Vorteil von PROMs besteht darin, dass sie nicht auf ärztliche Fremdeinschätzungen (Clinician-reported Outcomes, CRO) angewiesen sind. Dadurch lassen sich Aspekte abbilden, die für den Behandlungserfolg von Relevanz sind, aber in klassischen Endpunkten oft unzureichend berücksichtigt werden – zum Beispiel eine Schmerzbelastung, anhaltende Fatigue oder Funktionseinschränkungen im Alltag.

**Abb. 1:**
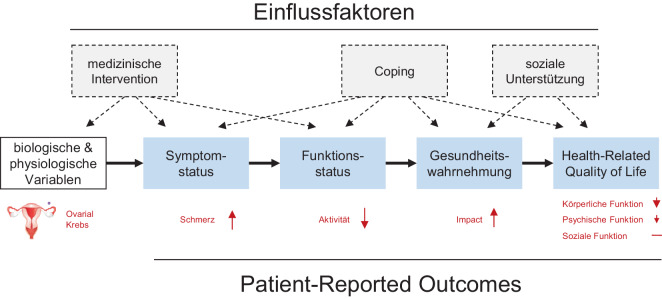
Theoretisches Model (Four Box Model) zum Zusammenhang zwischen körperlichen Veränderungen und verschiedenen Patient-reported-Outcome-Ebenen (modifiziert nach Wilson und Cleary [5])

Zudem hat sich eine inhaltliche Zuordnung verschiedener Konstruktebenen als sinnvoll erwiesen. In dem häufig zitieren Konzept von Wilson und Cleary ([5]; Abb. 1) gehen die Autoren davon aus, dass bei einer biologischen Intervention primär auf die Verbesserung somatischer Bedingungen abgezielt wird. So ist anzunehmen, dass zunächst die wahrgenommenen Symptome und die Funktionsfähigkeit günstig beeinflusst werden. Die individuelle Bewertung dieser Symptome und Funktionen fließt dann in das übergeordnete Konstrukt der empfundenen Gesundheit ein, was seinerseits Teil der Lebensqualität ist. Das heißt, die Relevanz der Erkrankung für die Lebensqualität hängt sowohl von biologischen Faktoren ab als auch von emotionalen Bewältigungs- und kognitiven Bewertungsvorgängen, die ihrerseits von einer Vielzahl kultureller und persönlicher Faktoren beeinflusst werden.

Zu der Messung der PREs findet sich in diesem Heft eine gesonderte Abhandlung, sodass im Folgenden der Fokus auf die Erhebung von PROs gelegt wird. Dafür wird auf die verschiedenen PROM-Nutzergruppen, die Auswahl eines geeigneten Instruments und die Geschichte der PRO-Nutzung eingegangen. Zudem werden der potenzielle Nutzen und verschiedene Gründe für eine zögerliche Implementierung in die klinische Routine erläutert.

## Nutzergruppen

**Abb. 2:**
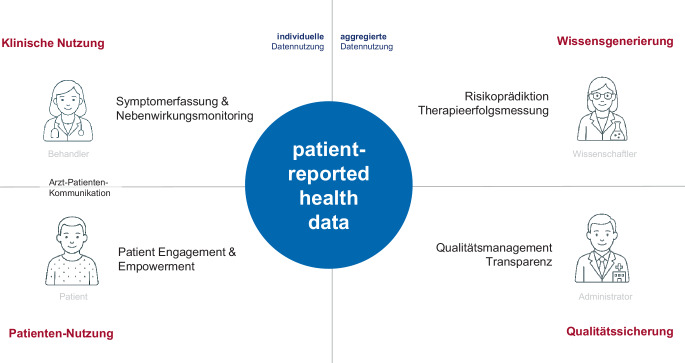
Vier Nutzergruppen von Patient-reported Outcome Measures (PROMs) und ihre Motive

Vereinfachend lassen sich 4 Gruppen unterscheiden, die unterschiedliche Motive für die PROM-Nutzung haben (Abb. 2). Für *Behandler* steht in der Regel die Identifikation psychischer Belastungen und Störungen [6] oder das Symptommonitoring im Vordergrund [1]. Für *Patienten* bieten digitale PRO-Systeme die Option, ihren Beschwerdeverlauf zu erfassen, etwa um Faktoren zu identifizieren, die sich günstig auf die Beschwerden auswirken, oder direkt mit ihren Behandlern zu kommunizieren. Für den *wissenschaftlichen Nutzer* erlauben PROMs u. a. die Effekte verschiedener Interventionen aus Patientensicht zu unterscheiden, während die *Qualitätssicherung* eher auf einen Vergleich verschiedener Akteure abzielt [7]. Die psychometrischen, methodischen und technischen Anforderungen an die PRO-Erfassung sind je nach Nutzung sehr unterschiedlich.

## Instrumente

Es liegt eine enorme Menge validierter Fragebögen vor, mit denen sowohl allgemeine Aspekte der Gesundheit als auch spezifische Symptome und Funktionen erfasst werden können. Die ePROvide-Datenbank des Mapi Research Trust listet aktuell über 8000 Instrumente auf, davon allein über 300 Instrumente zur Erfassung von Depressivität. Damit ist die Auswahl eines geeigneten Instrumentes häufig schwierig. Meist wird auf etablierte Instrumente zurückgegriffen, für die bereits Vorerfahrungen existieren, unter Umständen ohne zu prüfen, ob das Instrument tatsächlich dem Bedarf des Nutzers entspricht.

Allgemein kann ein Instrument nach verschiedenen Kriterien beurteilt werden, z. B. ob es *objektiv* ist (d. h. unabhängig von der Erhebungssituation), *reliabel* (d. h., ob es verlässliche Ergebnisse liefert) oder *valide* (d. h., ob die Ergebnisse sinnvoll interpretierbar sind). Für die Darstellung der Reliabilität wird häufig der Cronbach-α-Wert erwähnt, der die Kohärenz der Items einer Skala illustriert (*Internal Consistency*). Für die Validität einer Skala ist gleichzeitig jedoch eine hinreichende Repräsentanz der Facetten eines Konstrukts nötig, sodass eine gewisse Heterogenität der Items in der Regel sinnvoll ist (*Content Balancing*).

Daneben gibt es eine Reihe von Nebengütekriterien, u. a. wie ökonomisch (Anzahl der Items für die Erfassung eines Konstrukts), fair (gleiche Testeigenschaften für verschiedene Bevölkerungsgruppen) oder nützlich (lassen sich Entscheidungen auf der Grundlage der Ergebnisse treffen) ein Test ist. In der Praxis spielt zusätzlich eine Rolle, ob das Instrument frei verfügbar ist, Lizenzgebühren anfallen, die Integration in eine bestimmte Softwarelösung erlaubt ist oder Fachgesellschaften sich bereits auf ein spezifisches Instrument festgelegt haben.

Wenn man exemplarisch verschiedene Instrumente zur Erfassung von Depressivität gegenüberstellt, besteht die Möglichkeit, neben vielen anderen eines der folgenden zu nutzen: Becksches Depressionsinventar ([8]; BDI, 21 Items), Patient Health Questionnaire 9 ([9]; PHQ‑9, 9 Items), PHQ‑8 ([10]; 8 Items), den Wohlbefindens-Index der Weltgesundheitsorganisation ([11]; WHO‑5, 5 Items), PHQ‑2 ([12]; 2 Items) oder einen Computer Adaptiven Test (CAT) des Patient-reported Outcome Measurement Information Systems (PROMIS; [13]; ca. 2–6 Items).

So könnte ein Anwender das Instrument präferieren, was von den genannten am längsten etabliert ist, wie das BDI, das seit 1961 genutzt wird. Dieses ist jedoch relativ lang, es fallen Lizenzgebühren an und die Nutzung in digitalen Erfassungssystemen erfordert Vereinbarungen mit den Rechteinhabern. Andere könnten den PHQ‑9 wählen, der die ICD-10-Kriterien der Depression abbildet, frei verfügbar ist und für den bereits über 100.000 Publikationen vorliegen, bzw. den PHQ‑8, wenn aus rechtlichen Gründen auf die Erfassung der Suizidalität verzichtet werden möchte.

In der Diabetologie wird eher der WHO‑5 genutzt, der auch Depressivität misst, aber die Items positiv formuliert, was die Akzeptanz in der Diabetologie erhöht. Von Tumorpatienten wiederum kann dies als situationsinadäquat empfunden werden. Der PHQ‑2 eignet sich zum Screening oder für große Stichproben, ist aufgrund ungenügender Messpräzision für eine individuelle Verlaufsbeobachtung jedoch ungeeignet. CATs [13] versprechen einen besseren Kompromiss aus Testlänge, Messpräzision und Messbereich, erfordern jedoch deutlich höhere technische Voraussetzungen. Das heißt, die Auswahl eines passenden Instruments ist neben den psychometrischen Eigenschaften von einer Vielzahl anderer Faktoren abhängig.

Während anhand des Beispiels Depression verschiedene Instrumente gegenübergestellt werden, die alle ein gemeinsames Konstrukt mit ähnlichen Items erfassen (Domain Score), sind in anderen Feldern, wie der Onkologie und Kardiologie, Instrumente verbreiteter, die unterschiedliche Symptome einer Erkrankung zusammenfassen (Composite Score).

Bei einem *Domain Score* wird davon ausgegangen, dass es ein latentes Konstrukt gibt (z. B. Depressivität), was die Antworten auf die Einzelitems (z. B. Interessensverlust, Niedergeschlagenheit) gemeinsam beeinflusst. Bei einem *Composite Score* werden hingegen unterschiedliche Konstrukte zu einem Score zusammengefasst, die typischerweise durch einen externen Faktor beeinflusst werden. So kombiniert der Kansas City Cardiomyopathy Questionnaire (KCCQ-12; [14]) u. a. 3 Items zur Erfassung der körperlichen Funktion und 2 Items zur Atemnot mit je einem Item zu Müdigkeit und Beinödemen. Die Anwendung solcher Composite Scores ist nur für den Einsatz in spezifischen Gruppen sinnvoll, hier Patienten mit Herzinsuffizienz, während die domainorientierten Instrumente allgemeingültige Konstrukte erfassen, die erkrankungsunabhängig einsetzbar sind.

Häufig setzen sich in einzelnen Feldern spezifische Instrumente durch, obgleich ein bestimmtes Konstrukt in verschiedenen Situationen vermutlich durch unterschiedliche Instrumente besser messbar wäre. Dabei lässt sich ein unterschiedlicher Umgang der Rechteinhaber mit der Nachfrage nach einem Instrument erkennen. So ist der PHQ‑9 [9] ohne jedwede Restriktionen frei und kostenlos verfügbar. Ein Großteil der in der Onkologie eingesetzten Instrumente wird von der EORTC zur Verfügung gestellt. Diese ist eine gemeinnützige Organisation, die den Einsatz der EORTC-Instrumente für akademische Nutzung und nichtkommerzielle klinische Anwendungen in der Regel kostenlos ermöglicht, während für die kommerzielle Nutzung, z. B. durch die pharmazeutische Industrie, Lizenzgebühren anfallen. Nach dem gleichen Prinzip verfährt die EuroQol (EQ) und das deutsche PROMIS National Center, das vom Berlin Institute of Health (BIH) über Bundesmittel finanziert wird. Die Instrumente, die in der Kardiologie verbreitet sind, wie z. B. der KCCQ-12 [14] oder Atrial Fibrillation Effect on Quality-of-Life-Score (AFEQT; [15]), werden hingegen von kommerziellen Anbietern vertrieben, sodass auch für die akademische und klinische Nutzung regelhaft Lizenzgebühren anfallen.

## Historie

**Abb. 3:**
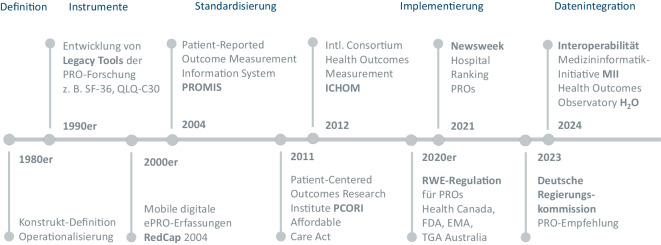
Historische Entwicklung der Erfassung generischer Patient-reported Outcomes (PROs). SF-36: Fragebogen zur Erfassung des Gesundheitsstatus – Short Form 36 Items; QLQ-C30: Quality of Life Questionnaire – Cancer 30 Items; *RedCap* digitales Erfassungssystem für PROs; *RWE* Real World Evidence; *FDA* Food and Drug Administration, *EMA* European Medicine Agency; *TGA* Therapeutic Goods Administration

Die wissenschaftliche Nutzung von PROs ist seit gut 5 Jahrzehnten gängige Praxis. Dabei erfolgten erste Pionierarbeiten überwiegend in dem Feld psychischer Gesundheit. So wurde z. B. das BDI bereits 1961 veröffentlicht [8]. Überlegungen zur Operationalisierung der Lebensqualität folgten deutlich später. Seit den 1980er-Jahren besteht Konsens, den gesundheitsbezogenen Teil der Lebensqualität (Health-related Quality of Life, HRQL) als mehrdimensionales Konstrukt zu verstehen, das sowohl physische, psychische und soziale Aspekte umfasst (Abb. 3). In den 1990er-Jahren stand die Instrumentenentwicklung im Vordergrund, die die HRQL-Domänen weiter differenzierte, z. B. des SF-36 [4], Core Quality of Life Questionnaire (QLQ-C30; [16]), WHO Quality of Life Assessment (WHOQOL; [17]), EQ-5D [18]. Seit der Jahrtausendwende steht eher die Harmonisierung oder *Standardisierung* im Fokus. So werden zunehmend Methoden entwickelt, wie die Ergebnisse ähnlicher Fragebögen ineinander umgerechnet werden können [19].

2004 wurde gemeinsam von allen National Institutes of Health (NIHs) in den USA die Entwicklung eines modularen PRO-Systems (PROMIS [20]) initiiert und damit ein Schritt in Richtung einer instrumentenunabhängigen, domainorientierten Messung gegangen. Ziel war, auf der Grundlage der probabilistischen Testtheorie eine Metrik zu schaffen, um mit verschiedenen Instrumenten eine spezifische Domäne, z. B. Depressivität, auf einer gemeinsam definierten Skala (Common Metric) interoperabel zu messen [21, 22]. Damit kann die Erfassung psychischer Konstrukte der Messung biologischer Parameter ähnlicher werden – analog der Temperaturmessung, die mit unterschiedlichen Thermometern auf einer gemeinsamen Temperaturskala erfolgt.

Vor mehr als 10 Jahren wurde das International Consortium for the Harmonization of Outcome Measures (ICHOM) gegründet, das bislang Harmonisierungsvorschläge zu über 40 Erkrankungsbildern vorgelegte [23]. Unabhängig hiervon haben auch einzelne Fachgesellschaften begonnen, sich auf gemeinsame Outcome-Domänen festzulegen, wie z. B. die OMERACT-Initiative in der Rheumatologie [24] oder die SONG-Initiative in der Nephrologie [25].

Derzeit ist die Entwicklung zunehmend mit den Herausforderungen der *Implementierung* beschäftigt [26]. Mit der Berücksichtigung von PROs bei der Klinikbewertung (*Newsweek Journal*), den Empfehlungen der Bertelsmann Stiftung [27] oder der Regierungskommission in Deutschland [28] wurde eine größere Öffentlichkeit für das Thema erreicht. Im Jahr 2025 hat der National Health Service (NHS) in Großbritannien seinen „Fit for the Future Plan“ vorgelegt, in dem der universelle Einsatz von PROMs und Patient-reported Experience Measures (PREMs) für alle Patienten bis 2029 vorgesehen ist. Das Deutsche Institut für Qualitätssicherung und Transparenz (IQTIG) entwickelt seit 2015 eigene PRO-Strategien, während die Deutsche Rentenversicherung [29] für die Weiterentwicklung ihres Qualitätssicherungsverfahrens (§ 37 Sozialgesetzbuch IX) den Fokus auf eine internationale Vergleichbarkeit legt und wie die Organisation für wirtschaftliche Zusammenarbeit und Entwicklung (OECD; PaRIS Survey [30]) u. a. die PROMIS-Metriken in aktuellen Studien nutzt.

## Nutzen

Bei fast 50 % aller klinischer Studien ist die Beurteilung der Intervention aus Perspektive der Patienten heute ein primäres oder sekundäres Erfolgskriterium [31]. Alle maßgeblichen Regulationsbehörden, wie das Institut für Qualität und Wirtschaftlichkeit im Gesundheitswesen (IQWiG), die European Medicines Agency (EMA) oder FDA, erwarten heute für die Nutzenbewertung neuer Medikamente den Einsatz validierter PROMs [2, 32]. Hinzu kommt, dass durch neue europäische Health-Technology-Assessment-(HTA-)Regulationen die pharmazeutischen Hersteller zusätzlich angehalten werden, den patientenzentrierten Nutzen auch nach der Zulassung durch Beobachtungsdaten zu belegen. Damit ist in den nächsten Jahren ein weiter steigendes Interesse an PRO-Daten aus der klinischen Routine zu erwarten.

Zudem kann die empirische Erfassung des Gesundheitszustands auch unmittelbaren, klinischen Nutzen entfalten [33]. Früh wurde bereits ein positiver Effekt für die Kommunikation zwischen Patienten und Behandlern gezeigt [34]. Die systematische Erfassung von Symptomen verhindert, Begleitsymptome zu übersehen, wie z. B. das Bestehen einer komorbiden Depressivität. Zudem ist der selbstberichtete körperliche oder psychische Gesundheitszustand von prädiktiver Bedeutung für die Mortalität bei onkologischen ebenso wie bei kardiometabolischen Erkrankungen [35, 36]. Der DAK-Gesundheitsreport 2022 schätzt die Erhöhung des Sterberisikos für Patienten mit Herzerkrankungen bei gleichzeitig bestehender Depressivität oder Erschöpfung mit dem Faktor 1,5 ein, was dem Risiko einer bestehenden Adipositas oder Hypercholesterinämie entspricht [37]. Für das Erkennen von individuellen Risiken kann die Nutzung von PROs daher eine sinnvolle und kostengünstige Hilfestellung bieten.

Bei der Behandlung psychischer Erkrankungen ist das Monitoring des subjektiven Erlebens selbsterklärend, aber auch in der Onkologie bekommt die empirische Erfassung von Symptomen zunehmend Bedeutung. So zeigen alle größeren Studien, dass mit einem Symptommonitoring die HRQL durch eine bessere Symptomkontrolle signifikant verbessert werden kann [38, 39]. Zudem haben einige Monocenterstudien auch positive Effekte auf die Überlebenszeit berichtet. Es ließ sich durch die ambulante Symptomerfassung sowohl die Zahl der Besuche in den Notaufnahmen reduzieren als auch die 5‑Jahres-Überlebenswahrscheinlichkeit um bis zu 6 % steigern [40]. Eine weitere Studie zeigte, dass die PRO-Erfassung zu einer schnelleren Anpassung der Therapie beiträgt und so die mediane Überlebenszeit bei Patienten mit fortgeschrittenen Lungentumoren von 12 auf 19 Monate verlängern konnte [41]. Eine 2025 veröffentlichte Multicenterstudie an einem gemischten Kollektiv konnte positive Effekte bezüglich der Beeinflussung der Lebensqualität bestätigen, zeigte allerdings keine Verlängerung der Lebenszeit [42]. Den Wert von PROs für die Qualitätsverbesserung hat insbesondere die Martini-Klinik demonstriert: Über eine systematische PRO-Erfassung sexueller Dysfunktion und Inkontinenz nach Prostata-OP sowie kontinuierliche Rückmeldungsschleifen konnte die Qualität des Operationserfolges drastisch gesteigert werden [43].

## Implementierung

Obwohl der potenzielle Nutzen von PROMs für eine patientenzentrierte Versorgung anerkannt ist, ist deren Nutzung in der klinischen Routine – abgesehen von einzelnen Pilotprojekten – weiterhin eher die Ausnahme [27]. Hierfür lassen sich verschiedene Gründe nennen.

### Harmonisierung und Standardisierung

Für eine patientenzentrierte Versorgung wäre eine Erfassung des selbstberichteten Gesundheitsstatus über alle Sektoren hinweg wünschenswert, einerseits, um den individuellen Behandlungsprozess besser steuern zu können, andererseits, um die Langzeiteffekte alternativer Therapien in der Praxis besser erkennen und vergleichen zu können. Hierfür ist ein Konsens über die erfassten Merkmale eine der Voraussetzungen. Die ICHOM-Initiative und verschiedene Fachgesellschaften haben über die letzte Dekade Vorschläge für eine derartige Standardisierung vorgelegt, die jedoch wenig aufgegriffen wurden. Ausschlaggebend hierfür könnte sein, dass die empfohlenen Fragebögen in der Regel für Forschungsfragestellungen entwickelt wurden und sich aufgrund ihres Umfangs für einen Einsatz in der klinischen Routine kaum eignen.

Die beschriebene Entwicklung hin zu einem domainorientierten Messsystem könnte sowohl für die Harmonisierung als auch für die klinische Akzeptanz Bedeutung haben [44]. So erscheint eine Einigung auf interessierende Konstrukte, wie z. B. Fatigue oder soziale Teilhabe, einfacher als eine Einigung auf spezifische Instrumente. Dies ermöglicht gleichzeitig auch den Einsatz moderner, modularer Messverfahren, die bei geringer Belastung eine höhere Präzision über einen größeren Messbereich bieten [45], sowie die Möglichkeit, die Instrumente auf die jeweilige Situation anpassen zu können, z. B. für Personen mit einem geringen Sprachverständnis oder ältere Personen (Tailored Assessment).

**Abb. 4:**
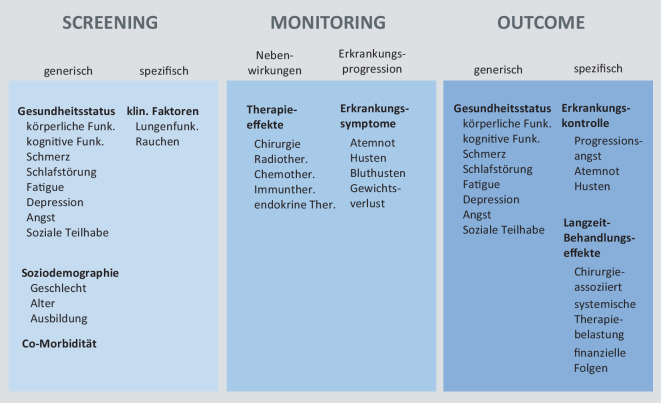
Beispiel eines domainorientierten Messmodells von Patient-reported Outcomes beim Lungentumor. Die initiale Erfassung erfolgt einmalig, das Monitoring mit wenigen Items abhängig vom klinischen Bedarf, die Erfassung der Outcomes meist ein- bis 4‑mal pro Jahr. *Funk.* Funktion; *klin.* klinisch; *Ther.* Therapie

International wie national sind Initiativen entstanden, die aus Sicht einzelner Fachgruppen hieran arbeiten. So scheint sich ein Konsens abzuzeichnen, für die Erfassung der HRQL ca. 8 Domänen zu betrachten (Abb. 4), wie sie größtenteils, z. B. im SF-36, QLQ-C30 oder PROMIS-29, erfasst werden. Durch die domainorientierte Messung können die Anwender das individuell präferierte Instrument nutzen, wobei die Scores auf einer gemeinsamen Metrik berichtet werden können. Dies kann mit je 2 Items pro Domäne (z. B. PROMIS-16) oder mehr erfolgen, je nach Anforderung. Auf dieser Grundlage lassen sich bekannte übergeordnete Maße wie der Physical und Mental Component Score des SF-36 (PCS/MCS; [46]) ebenso wie Utility Maße (z. B. PROMIS Preference Score (PROPR) oder EQ-5D [47]) ohne Mehraufwand berechnen. Für das Monitoring wird z. B. in der Onkologie empfohlen, eine individuelle Auswahl von Items aus der PRO Common Terminology Criteria for Adverse Events (PRO-CTCAE; [48]) abhängig von Erkrankung und Therapie zusammenzustellen.

### Digitale Lösungen

In vielen Gesundheitseinrichtungen sind Dokumentationsprozesse bereits sehr komplex. Werden PRO-Erhebungen zusätzlich angeboten, ohne dass eine Integration in bestehende Prozesse erfolgt, droht eine inakzeptable Mehrbelastung des Personals. Fehlende Schnittstellen zum Krankenhausinformationssystem gehören daher zu den am häufigsten genannten Hemmnissen. Bei vielen etablierten PRO-Erfassungssystemen, wie z. B. RedCap, wird zudem kaum Augenmerk auf die individuelle Darstellung der PRO-Ergebnisse für die klinische Ergebnisinterpretation gelegt. Eine grafische Rückmeldung an die Patienten fehlt oft ganz.

**Abb. 5:**
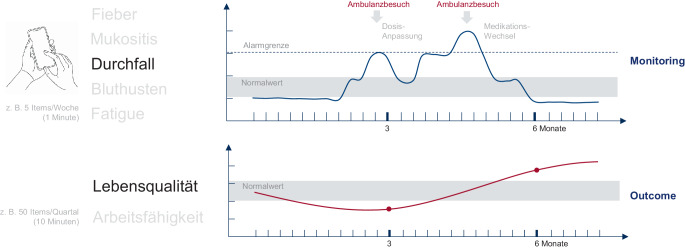
Beispiel der Kombination von Symptom- und Ergebnismessung bei einem individuellen Patienten, wie es für das Nationale Centrum für Tumorerkrankungen derzeit entwickelt wird

Eine gelungene Integration von PROMs in den klinischen Alltag wird daher maßgeblich durch die Fortschritte der Digitalisierung geprägt sein [26]. Elektronische Erhebungsverfahren – etwa über mobile Anwendungen, Tablets in Kliniken oder Patientenportale – ermöglichen bereits heute eine effizientere Datenerhebung. So könnte eine ambulante Symptomerfassung mit wenigen Items in kurzen Intervallen erfolgen und mit der Erfassung tradierter Fragebögen in größeren Intervallen verbunden werden. Abb. 5 illustriert ein Beispiel aus der Onkologie, wie es für das Nationale Centrum für Tumorerkrankungen (NCT) in Zukunft zur Verfügung stehen wird. Dabei sollen handlungsrelevante Ergebnisse in einfacher Form dargestellt werden, sodass auf auffällige Werte gezielt reagiert werden kann. Einige Beispiele hierfür sind bereits in den klinischen Alltag übernommen worden, z. B. bei der Nachverfolgung von Patienten nach Nierentransplantation.

### Datenintegration

Das Interesse an der Erfassung von PROs in der klinischen Praxis dürfte zusätzlich steigen, wenn die Verknüpfung einer primären Nutzung für die individuelle Behandlung mit einer sekundären Nutzung der Daten für wissenschaftliche Fragestellungen oder zur Qualitätssicherung gelingt.

Die Medizininformatik-Initiative (MII; [49]) arbeitet derzeit in verschiedenen Projekten (z. B. PCOR-MII) u. a. an der Integration von PROs in deren Kerndatensatz, um eine interoperable PRO-Erfassung in allen deutschen Universitätskliniken zu ermöglichen.

Europaweit hat die exemplarische Entwicklung eines paneuropäischen Health Outcome Observatory (H_2_O) begonnen [50], was für einige Entitäten demonstrieren soll, wie auf internationaler Ebene patientenberichtete Gesundheitsmerkmale zur Verbesserung der Versorgung genutzt werden können. Langfristiges Ziel ist, PRO-Daten auch für den European Health Data Space (EHDS) verfügbar zu machen. Falls diese Initiativen sich erfolgreich weiterentwickeln, könnte eine empirisch basierte, stratifizierte Behandlung möglich werden, die neben biomedizinischen Parametern auch psychosoziale Merkmale des Patienten berücksichtigt und als Behandlungsziel das Wohlbefinden der Patienten in großem Umfang messbar macht.

## Diskussion und Fazit

Die Erfassung von PROs markiert einen Paradigmenwechsel im Gesundheitswesen. Während lange Zeit vor allem objektive Kennzahlen wie Mortalität, Morbidität oder Prozessindikatoren im Vordergrund standen, rückt nun zunehmend die Sichtweise der Patienten selbst ins Zentrum. PROs ermöglichen es, Behandlungsergebnisse jenseits klinischer Surrogatmarker zu erfassen und damit die Wirksamkeit von Interventionen aus der Perspektive der Betroffenen sichtbar zu machen.

Über die letzten Jahrzehnte wurde eine große Zahl validierter Fragebögen entwickelt, die eine differenzierte Erfassung einer Vielzahl von Konstrukten erlaubt, mit denen der potenzielle Wert der PRO-Messung sowohl für den Wissenszuwachs als auch für die individuelle Behandlung gezeigt werden konnte. In verschiedenen Studien wurde demonstriert, dass sich PROs für die Identifikation von individuellen psychischen Belastungen, komorbiden Erkrankungen und die Risikoprädiktion ebenso eignen wie für eine systematische Erfassung positiver und negativer Effekte medizinischer Therapien.

Die Erfassung von PROs erweist sich in der Onkologie zudem selbst als Intervention mit durchgängig positiven Effekten auf die Lebensqualität und in einigen Studien sogar auf die Überlebenszeit der Erkrankten durch eine verbesserte Therapiesteuerung und -adhärenz. Trotz ihres Potenzials bei relativ geringen Kosten findet eine systematische Erfassung von patientenberichteten Gesundheitsmerkmalen in der klinischen Praxis bislang nur in Ausnahmefällen statt. Die Gründe für die Zurückhaltung bei der Implementierung von PRO-Erfassungssystemen in der klinischen Praxis könnten zum einen in der Methodik selbst liegen, zum anderen an einer mangelnden Integration in die klinischen Prozessabläufe.

Ein Großteil der Fragebögen, die in der wissenschaftlichen Nutzung besonders verbreitet sind, wurde primär für den Einsatz in klinischen Studien entwickelt und ist aufgrund ihres Umfangs und geringer Anpassungsmöglichkeiten für den Einsatz in der Routine wenig geeignet. Zudem sind für wichtige Konstrukte, wie z. B. die Erfassung der Depressivität, eine große Zahl von ähnlichen Fragebögen etabliert, was eine Harmonisierung der Erfassung über verschiedene Anwendungsfelder erschwert. Internationale Initiativen haben bislang auf eine Standardisierung der Erfassung der Ergebnismessung (*Outcome*) fokussiert. Für die Akzeptanz in der Praxis erscheint die Nutzung von PROs für die Identifikation individueller Belastungen oder Erkrankungen, die Erfassung spezifischer Risiken (*Screening*) oder für die Therapiesteuerung (*Monitoring*) jedoch weitaus interessanter. Enthalten PRO-Daten keine handlungsrelevanten Informationen oder werden primär für externe Zwecke erhoben, etwa für die Zertifizierung oder die öffentliche Berichterstattung, sinkt die Motivation für deren Nutzung im klinischen Alltag.

Um die individuelle Primärnutzung von PRO-Daten mit einer aggregierten Sekundärdatennutzung zu verbinden, sind in den letzten Jahren modulare Erfassungssysteme entstanden, die versprechen, zentrale Konstrukte der HRQL instrumentenunabhängig zu erfassen. So erscheint es wahrscheinlicher, sich auf eine intersektorale Erfassung bestimmter Domänen zu einigen als auf die Nutzung spezifischer Instrumente.

Methoden der probabilistischen Testtheorie erlauben die Erfassung eines Konstrukts mit verschiedenen Items auf einer gemeinsamen Metrik. Damit wird es u. a. möglich, die Messung auf die individuellen Bedingungen anzupassen, z. B. Personen mit geringerem Sprachverständnis einfachere Items vorzulegen oder für einmalige Messungen umfangreichere Instrumente einzusetzen als für eine häufigere Erfassung – ohne die Vergleichbarkeit infrage zu stellen. Damit würde die Erfassung von PROs der Messung biomedizinischer Parameter ähnlicher werden, sodass auch die Akzeptanz für deren klinische Nutzung steigen könnte. Von herausgehobener Bedeutung erscheint, in diesem Zusammenhang zeitnah eine modulare therapiespezifische Symptomerfassung zu etablieren. Hiermit scheint es möglich, z. B. in der Onkologie Nebenwirkungen effektiv zu kontrollieren und so positive Effekte auf die Therapieadhärenz zu erreichen.

Eine weitere Voraussetzung für die Nutzung von PROs in der klinischen Routine sind eine adäquate Datenerfassung und -verarbeitungsmöglichkeit. Je besser die digitale PRO-Lösung in die Routineabläufe integriert ist, desto höher ist die Chance für eine erfolgreiche Nutzung. Eine Reduktion der Ergebnisdarstellung auf wenige handlungsrelevante Informationen ist dabei notwendig.

Insgesamt sollte die Einführung in die klinische Praxis den unmittelbaren klinischen Nutzen im Fokus haben, dem die sekundäre Datennutzung für wissenschaftliche Fragestellungen und die Qualitätsverbesserung nachgeordnet sind. Ohne sorgfältige Implementierungsstrategien werden PROMs von den meisten Anwendern bislang eher als zusätzliche Belastung wahrgenommen, statt ihren Mehrwert zu entfalten [26].

International betrachtet zeigen Beispiele aus England, Skandinavien und den USA, dass PROs auch in großem Maßstab implementierbar sind und wegweisend für eine patientenzentrierte Versorgung sein können. In Frankreich wird das Symptommonitoring in der Onkologie von den Kassen bereits vergütet und landesweit eingesetzt. Insbesondere Deutschland hat in den letzten Jahren langfristige Initiativen angestoßen, die für die PRO-Datenintegration impulsgebend sein können. Sollte die transsektorale Harmonisierung u. a. im NCT, der MII bzw. dem Netzwerk Universitätsmedizin (NUM) gelingen und in den EHDS überführt werden, könnte ein europäischer Datenraum entstehen, der für die patientenzentrierte Versorgung und Forschung weltweit einzigartig wäre.

Zusammenfassend lässt sich festhalten, dass PROs trotz der beschriebenen Schwierigkeiten in den kommenden Jahren von punktuellen Projekten hin zu einem festen Bestandteil der Routineversorgung in einzelnen Fachgebieten werden dürften. Langfristig scheint kaum denkbar, dass eine wissenschaftlich basierte Medizin ohne empirisch fundierte Kenntnis darüber, wie es den Behandelten geht, auskommen wird.

## Data Availability

Für die Erstellung der Arbeit wurden keine Daten verarbeitet.
